# Identification of a novel methyltransferase-type 12 protein from *Haemonchus contortus* and its effects on functions of goat PBMCs

**DOI:** 10.1186/s13071-020-04028-y

**Published:** 2020-03-30

**Authors:** Muhammad Ehsan, Javaid A. Gadahi, Tingqi Liu, Mingmin Lu, Yujian Wang, Muhammad W. Hasan, Muhammad Haseeb, Ruofeng Yan, Lixin Xu, Xiaokai Song, Xing-Quan Zhu, Xiangrui Li

**Affiliations:** 1 State Key Laboratory of Veterinary Etiological Biology, Key Laboratory of Veterinary Parasitology of Gansu Province, Lanzhou Veterinary Research Institute, Chinese Academy of Agricultural Sciences, Lanzhou, 730046 Gansu People’s Republic of China; 2 MOE Joint International Research Laboratory of Animal Health and Food Safety, College of Veterinary Medicine, Nanjing Agricultural University, Nanjing, 210095 Jiangsu People’s Republic of China; 3 Department of Veterinary Parasitology, Sindh Agriculture University Tandojam, Hyderabad, Pakistan

**Keywords:** *Haemonchus contortus*, Methyltransferase-type 12, PBMC, Goat, Host–parasite interactions

## Abstract

**Background:**

Methyltransferases (MTFs) are broad range of enzymes, which are ubiquitously expressed in diverse organisms ranging from bacteria to animals. MTFs proteins have been associated with various biological/cellular processes including transcriptional regulation, subcellular protein and RNA localization, signal transduction and DNA-damage repair. However, the role of MTFs in immune mechanism during host–parasite interaction has not been addressed yet.

**Results:**

An open reading frame (764 bp) of methyltransferase-type 12 gene of *H. contortus* denoted as HcMTF-12, was successfully cloned using reverse transcriptase-polymerase chain reaction (RT-PCR) followed by prokaryotic expression in *Escherichia coli* BL21 (DE3 strain). The recombinant HcMTF-12 protein (rHcMTF-12) was about 47 kDa along with a fusion vector protein of 18 kDa. Immunoblot results identified the native protein MTF-12 with antibodies produced in rats against rHcMT-12, whereas rHcMTF-12 protein was recognized with sera of goat experimentally infected with *H. contortus*. Immunohistochemical analysis revealed that the native MTF-12 protein was mainly located in the periphery (cuticle) of parasite sections as well as within the pharynx and intestinal region. An immunofluorescence assay validated that rHcMTF-12 attached to the surface of goat PBMCs. Furthermore, the cytokines transcription of IL-2, IFN-γ and IL-4 transcripts of PBMCs incubated with rHcMTF-12 were enhanced in a dose-dependent manner. The secretion of TGF-β1 and IL-10 was significantly decreased. However, IL-6 production was not significantly different as compared to the control groups. Moreover, the migration activity and nitric oxide (NO) production by PBMCs were induced considerably, whereas the proliferation of PBMCs cells was negatively affected when incubated with the rHcMTF-12 protein.

**Conclusions:**

Our findings suggest that HcMTF-12 significantly mediated the functions of PBMCs, and it might be a potential candidate for therapeutic interventions against haemonchosis.
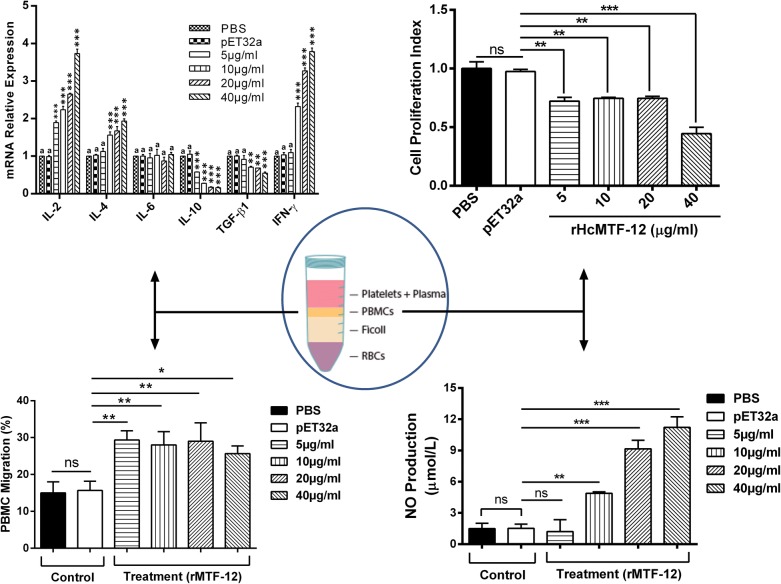

## Background

Methyltransferases (MTFs) are a broad range of enzymes, which are ubiquitously expressed in varied organisms ranging from bacteria to animals [1]. MTFs are membrane-bound enzymes that transfer a methyl group from S-adenosyl-l-methionine to thiol, amino or catechol acceptor molecules and this reaction modifies RNA, DNA and protein molecules for regulatory activities [2, 3]. Previously, more than 150 methyltransferase enzymes have been documented with structural differences but similar characteristics within the local active sites [4]. MTFs have been implicated in transcriptional regulation, subcellular protein and RNA localization, DNA-damage repair and signal transduction [5]. The multiple roles of methylated DNA in restriction-modification systems of prokaryotes and various cellular processes including gene differentiation and regulation in eukaryotes are well documented. However, apart from biochemical/structural properties, the role of methyltransferases participating in immune mechanism during host–parasite interaction has not been addressed yet. S-adenosyl-l-methionine (Ado-Met), a methyl donor in methyl transfer system has been suggested as an important pathway for metabolism of endogenous as well as xenobiotic compounds [6]. A previous study highlighted that methylation is an essential process to control gene transcription through which many drugs and small molecules undergo methylation, including nucleic acids, polysaccharides, lipids, proteins, hormones and neurotransmitters [7]. A recent research in the context of drug discovery and drug development revealed that MTFs are involved in using of S-Adenosyl methionine (SAM) analogs to methylate naturally produced anticancer agents which keeps alternate alkyl group as a substitute for methyl [8].

*Haemonchus contortus* is a parasite of veterinary importance globally by inducing huge economic and production losses due to severe anemia, hemorrhagic gastritis, oedema and even death of infected small ruminants [9]. After three successive developmental stages that occur in the environment, the third-stage (L3) larvae are taken into hostʼs digestive tract during grazing where fourth-stage larvae (L4) develop into mature adult worms feeding on blood from capillaries in the abomasum [10]. The widespread use of anthelmintic drugs as an effective method for control of *H. contortus* infections and generation of parasitic resistance to these commercially available drugs has become a serious problem worldwide [11–13]. During host–pathogen interactions, many proteins and other molecules of pathogens called excretory/secretory (ES) proteins target the host proteins to facilitate the infection process and modulate host immune response [14]. ES antigens of *H. contortus* possibly perform significant roles in infection process at the parasite-host interface [15].

Our previous proteomic analysis of *H. contortus* ES proteins bound to peripheral blood mononuclear cells (PBMCs) of goats revealed that methyltransferase type-12 is an important ES interacting antigen bound to goat immune cells at L4 and L5 developmental stages of *H. contortus in vivo* [16], indicating that this binding may have vital role in modulation of host immune responses during infection process. Here, we cloned and characterized the gene MTF-12 from *H. contortus* and describe the effects of the recombinant protein rHcMTF-12 on host PBMCs.

## Methods

### Experimental animals

*Haemonchus contortus*-free goats (local crossbreed) 3–6 months of age were housed indoors at the animal experimental station of Nanjing Agricultural University. Animals were given whole-shelled corn and hay, and water *ad libitum*. The goats have been dewormed with levamisole orally (8 mg/kg body weight) at a 2 week dose gap. In accordance with standard parasitological techniques, faecal samples were examined microscopically twice a week for helminth infection and goats having no sign of parasitic infection were used for the functional study. Three biological repeats (three goats), each comprising of three technical repeats (three replicates for each goat), were used in immunological functional experiments such as cytokine transcriptional analysis, immunofluorescence assays, nitric oxide (NO) production, cell proliferation and migration assays.

Specific anti-protein antibodies were produced in female Sprague-Dawley (SD) (~ 150 g body weight) rats bought from the Experimental Animal Center of Jiangsu Province, PR China (certified: SCXK 2008-0004). The rats were kept in a microbe-free state.

### Parasite collection and mononuclear cell separation/culture

Helminth-free goats (6 months of age) from the research and teaching flock at Nanjing Agricultural University were administered orally with approximately 10,000 L3 larvae of *H. contortus* (Nanjing strain 2005). The microscopical examination of fecal samples was performed on a weekly basis for the existence of parasite infection. The collection and preservation of parasite eggs, larvae, and adult worms were performed in accordance with the previously described methods [17].

Blood samples were collected from dewormed healthy goats and cultured as previously described [18]. Briefly, whole blood was taken by venipuncture of the jugular vein into a 10 ml vacutainer coated with ethylenediaminetetraacetic acid (EDTA) and brought to laboratory for PBMCs isolation and culture. PBMCs were separated by Ficoll-hypaque (GE Healthcare, Munich, USA) using a density gradient centrifugation protocol [19]. After washing twice with phosphate-buffered saline (PBS: Ca^2+^/Mg^2+^-free, pH 7.4), cell viability assessed by the trypan blue exclusion test was > 95% in all experiments. The isolated PBMCs were then cultured in complete medium RPMI 1640 (Roswell Park Memorial Institute 1640) or DMEM (Dulbeccoʼs Modified Eagleʼs Medium) containing 100 U/ml penicillin and 100 mg/ml streptomycin together with 10% heat-inactivated fetal bovine serum (FBS), (Gibco, Paisley, UK). Before performing cell isolation, strict precautions were taken and all solutions were passed through a syringe-supported Millex^®^GP 0.22 μm filter (Merck Millipore Ltd., Cork, Ireland) to avoid contamination. For functional analysis, PBMCs were cultured in 24-well flat bottomed culture plates (Costar, Cambridge, MA, USA) at 37 °C in 5% CO_2_. To investigate the effects of rHcMTF-12 protein on certain functions of goat PBMCs, cultured cells were subjected to rHcMTF-12 protein with discrete concentrations of 5 μg/ml, 10 μg/ml, 20 μg/ml and 40 μg/ml. PBMCs treated with empty-pET32a protein (10 μg/ml) and treated with PBS (pH 7.4) were used as controls in all triplicate experiments.

### RNA extraction and synthesis of 1st strand cDNA

Approximately 500 adult *H. contortus* parasites were crushed and homogenized at 4 °C in a pestle containing 1 ml Trizol (Invitrogen, Carlsbad, CA, USA), and total RNA was isolated under RNase-free conditions according to a previously described procedure [20]. Briefly, the homogenized mixture was transferred to sterile 2 ml Eppendrof tube and RNA was precipitated with the addition of 0.25 ml isopropyl alcohol per 1 ml of Trizol and centrifuged at 10,000×*g* for 10 min. After treatment with 70% ethanol in diethyl pyrocarbonate (DEPC), the pellet was finally dispensed in RNase/DNase free water and immediately used for cDNA synthesis. The RNA quantity was assessed using a spectrophotometry at optical density of 260 nm (OD260) and the quality was determined by the OD260/OD280 ratio. Only samples with an OD260/OD280 ratio between 1.8–2.0 were used. cDNA was synthesized using the PrimeScript^TM^ II 1st strand cDNA synthesis kit (TaKaRa Biotechnology, Shiga, Japan) as per the manufacturer’s instructions and stored at – 20 °C until further use [17].

### Cloning and characterization of HcMTF-12 gene

The open reading frame (ORF) of the *H. contortus* MTF-12 gene (GenBank/Uniprot: CDJ87424.1/ U6P1M9) was used to design the sense primer (5ʹ-GAA TTC ATG CGC AAG GAC GCT G-3ʹ) and anti-sense primer (5ʹ-CGA GCT CTT AAC CTG CAA CCA CTG TC-3ʹ) by using Primer Premier v5 software. The *EcoR*I and *Sac*I sites (underlined) were contained in the primers, respectively. The PCR reaction mixture (total volume of 50 μl) contained 50 μM 10× LA PCR buffer (Mg^2+^ Free) (TaKaRa Biotech, Dalian, China), 400 μM dNTP mixture, 3 mM MgCl_2_, 1 U Taq DNA polymerase (TaKaRa Biotech), 2 μl cDNA template and 400 nM forward and reverse primers. The PCR amplification cycles were as follows: 95 °C for 3 min followed by 40 cycles (at 95 °C for 35 s, 55 °C for 35 s, 72 °C for 60 s), followed by a final extension step at 72 °C for 8 min. The amplified PCR product was run on a 1% agarose G-10 (Biowest, Gene Co., Ltd., Chai Wan, Hong Kong) gel electrophoresis and purified with a gel purification kit (Omega, Norcross, GA, USA). The target fragment was ligated in the frame of the pMD-19T cloning vector (TaKaRa Biotech) followed by transformation into Trans5α competent cells (TransGen Biotech, Beijing, China). The amplified MTF-12 gene was subjected to restriction endonuclease digestion with *EcoR*I and *Sac*I, and sequenced by GenScript (Nanjing, Jiangsu, China). Subsequently, the identified target gene (HcMTF-12) was cloned into pET-32a (+) prokaryotic expression vector (Novagen, Madison, USA) to produce pET-32a (+)-HcMTF-12 and ligated into *E. coli* BL21. Positive clones were digested with *EcoR*I and *Sac*I and confirmed again by sequencing (GenScript, Nanjing, Jiangsu, China). The target gene was further subjected to bioinformatics analysis with available online sequences on GenBank (http://www.ncbi.nlm.nih.gov/BLAST/).

The comparison of the HcMTF-12 protein fragment with that of other parasites was carried out using BLASTx and BLASTp (https://blast.ncbi.nlm.nih.gov/Blast.cgi), and all sequences were aligned using ClustalW v1.8 software (http://www.clustal.org/). A phylogenetic tree was constructed using the neighbor-joining method in MEGA v6.0 program [21].

### Purification of *H. contortus* MTF-12 recombinant protein

The bacterial culture containing pET-32a (+)-HcMTF-12 was re-cultured in 1 l Luria Bertini (LB) medium containing ampicillin (100 μg/ml) for 2–3 h at 37 °C and 120× *g*. After the OD_600_ of the culture reached 0.6, 1 mM isopropyl-b-d-thiogalactopyranoside (IPTG; Solarbio, Beijing, China) was added to induce protein expression for 7 h and samples were taken at different time points at 1-h intervals to check the ascending expression level. The cells were collected by centrifugation at 4 °C and 8000×*g* for 20 min and washed twice with PBS. The pellet was lysed with 10 μg/ml lysozyme (Sigma-Aldrich, Shanghai, China) followed by sonication at 600 W for 30 min and centrifuged at 8000×*g* for 10 min at 4 °C. The rHcMTF-12 protein was eluted using a Ni^2+^-nitrilotriacetic acid (Ni-NTA) column (GE Healthcare). Thereafter, purified protein was assessed on 12% (w/v) sodium dodecyl sulfate polyacrylamide gel electrophoresis (SDS-PAGE) followed by staining with R-250 Coomassie brilliant blue (Sigma-Aldrich). The fused protein of pET-32a with the 109aa Trx·Tag™ thioredoxin protein and six histidines was extracted using the same procedure described for the recombinant protein. The endotoxin level in samples was quantified using a Limulus Amebocyte Lysate (LAL) gel clot assay using a Pyrosate® Kit (Cape Cod Inc., East Falmouth, MA, USA). Finally, the proteins were quantified using the Bradford method [22], according to the described procedure, and stored at − 20 °C for functional analysis.

### Antibody generation and confirmation by western blot

Polyclonal antibodies against rHcMTF-12 protein (anti-rHcMTF-12) were produced using 300 μg of rHcMTF-12 protein homogenized with an equivalent volume of Freund’s complete adjuvant injected intradermally into 2 female SD rats as described previously [23]. Two weeks after the primary dose, the rats were given three booster doses, seven days apart, using the same quantity and administration route as previously of rHcMTF-12 mixed with Freund’s incomplete adjuvant. Ten days after the last booster immunization, the rats were anesthetized with 25% isoflurane and sera with anti-rHcMTF-12 antibodies were separated from the blood and stored at − 20 °C for further applications. The sera against *H. contortus* infection were separated from blood of experimentally infected goats [17]. Sera separated from blood of normal goats/rats (un-immunized) were used as a control. The antibodies were quantified by ELISA.

For assessment of the specific reactivity with rHcMTF-12, an immuno-blot analysis was performed. Briefly, the rHcMTF-12 protein was separated on 12% SDS-PAGE and shifted onto Hybond^®^ ECL™ nitrocellulose membranes (Sigma-Aldrich). Unnecessary binding sites were prevented by 5% skimmed milk mixed with Tris-buffer saline containing 0.1% Tween-20 (TBST). The membrane was washed thrice (5 min per wash) in TBST, and further incubated with the primary antibody (1: 300 dilution in TBST) anti-rHcMTF-12 at 4 °C overnight followed by the secondary antibody (1: 3000 dilution in TBST), horseradish peroxidase (HRP)-conjugated goat anti-rat IgG (Sigma-Aldrich), for 2 h 37 °C. The reactions were visualized by exposing to 3, 3-diaminobenzidine tetrahydrochloride (DAB) (Boster Bio-technology).

### Immunofluorescence assay (IFA)

PBMCs from freshly collected goat blood were incubated with rHcMTF-12 protein (treatment group) or pET32a empty protein (positive control group) and PBS (negative control group) for 2 h at 37 °C with 5% CO_2_. PBMCs were fixed on poly-l-lysine-treated slides with 4% paraformaldehyde and permeabilized using 1% TritonX-100 in PBS. Thereafter, cells were treated with the primary antibody, rat anti-rHcMTF-12 IgG (1:300 dilution) or negative rat sera, followed by the secondary antibody (1:3000 dilution), Cy3-coupled goat anti-rat IgG (Beyotime Biotechnology, Shanghai, China) for 1 h at 37 °C in the dark. Cells were stained for 5 min in the dark with 1.5 μM 2-(4-amidinophenyl)-6-indolecarbamidine dihydrochloride (DAPI: Sigma-Aldrich) and visualized under a laser microscope (LSM710; Zeiss, Jena, Germany). Each step was performed after washing slides three times with PBS.

### Distribution of MTF-12 protein in worms

Adult male/female *H. contortus*, suspended in Tissue-Tek® OCT (Sakura, Torrance, CA, USA), were solidified in liquid nitrogen. The samples were sliced into 10 μm thickness using Cryotome (CM1950, Wetzlar, Germany), and allowed to settle on poly-l-lysine hydrobromide slides (Merck Millipore, Darmstadt, Germany). The slides were incubated with first and secondary antibodies, rat-anti-HcMTF-12 (1:300 dilution) or negative rat sera and Cy3-labeled goat anti-rat IgG (1:3000 dilution) (Beyotime Biotechnology), respectively, for 2 h at 37 °C. Cell nuclei were stained with DAPI (Sigma-Aldrich) and examined under a confocal microscope (LSM710; Zeiss).

### Cytokine transcriptions evaluated by real-time PCR

PBMCs were separated at a density of 1 × 10^6^ cells/ml using Ficoll® Hypaque Lymphocyte Separation Medium (GE Healthcare). Then cells were added into a 24-well plate (1 ml/well) containing concanavalin A (ConA: 10 μg/ml) and cultured with control buffer (PBS) and pET-32a control protein (10 μg/ml) or rHcMTF-12 protein (5, 10, 20 and 40 μg/ml) at 37 °C and 5% humidity for 24 h. After washing with PBS, the cells were subjected to total RNA extraction with PrimeScript^TM^ II 1st strand cDNA synthesis kit (TaKaRa Biotech) following the manufacturer’s guidelines. The quantity and quality of extracted RNA was analyzed using a spectrophotometer (Eppendorf, Hamburg, Germany) at absorption values between 1.8 and 2.0 (OD260/OD280). The primers used for cytokine detection (IL-2, IL-4, IL-6, IL-10, TGF-β1 and IFN-γ) including an endogenous reference gene (β-actin), their amplification efficiencies as well as standard reaction conditions, are provided in Additional file 1: Tables S1–S3. Transcriptional analysis was carried out on an ABI 7500 real-time PCR system (Applied Biosystems, Foster City, USA). The data were analyzed based on raw cycle thresholds (Cq), obtained from the ABI Prism 7500 software [24] using the comparative Cq method (2^−ΔΔ Cq^ method).

### Cell proliferation assay

Anti-proliferative efficiency of rHcMTF-12 was assessed by culturing goat PBMCs in a 96-well tissue culture plate (Corning Life Sciences, New York, USA) as described previously [25]. Briefly, 100 µl of cells (1 × 10^6^ cells/well) was activated with ConA and cultured with different rHcMTF-12 protein dilutions (5, 10, 20 and 40 µg/ml) or with pET-32a protein and PBS control, in triplicate. The mixture was cultured at 37 °C and 5% CO_2_ for 72 h, and 10 µl of CCK-8 solution (Beyotime Biotechnology) was mixed by pipetting in each well 4 h before harvesting. The optical density of the mixture was checked at 450 nm (OD_450_) using a spectrophotometer.

### Cell migration activity of PBMCs

Migration assessment of the cells was evaluated using sterilized 8.0 μm pore polycarbonate membrane inserts [26]. Briefly, 200 μl of 1 × 10^6^ cells in RPMI medium either rHcMTF-12-tretaed or untreated (control) were added into the Millicell® insert (Merck Millipore, Hayward, CA, USA) and placed on a Transwell tissue culture plate (Corning). Subsequently, the lower section was filled with 1300 μl of RPMI medium. The plate was incubated for 4 h in 5% CO_2_ at 37 °C. After incubation, PBMCs that moved through the membrane into the bottom of the well were determined using an automated cell counter (Countstar, Shanghai, China). The results are presented as percentages of the seeded PBMCs.

### Nitric oxide assay

One hundred µl of 1 × 10^6^ goat PBMCs per ml in DMEM medium were added into 96-well plate with varying concentrations of rHcMTF-12 (5, 10, 20 and 40 µg/ml) or pET32a and control buffer (PBS). After 48 h of cell culture at 37 °C and 5% CO_2_, the intracellular nitrite secretion of PBMCs was measured using the Griess assay [27] in accordance with total nitric oxide assay kit (Beyotime Biotechnology). Absorbance values were quantified on a spectrophotometer at 540 nm (OD_540_). Data were obtained from three independent experiments.

### Data analysis

Data were presented as the mean ± standard error of the mean (SE). Statistical analysis for significant differences were carried out using a one-way analysis of variance (ANOVA) using Graphpad Prism^TM^ v6 software (San Diego, California, USA), followed by a Tukeyʼs *post-hoc* test. The Student’s t-test was used for parametric samples and considered statistically significant at *P* < 0.05.

## Results

### Amplification and cloning of the HcMTF-12 gene

**Fig. 1:**
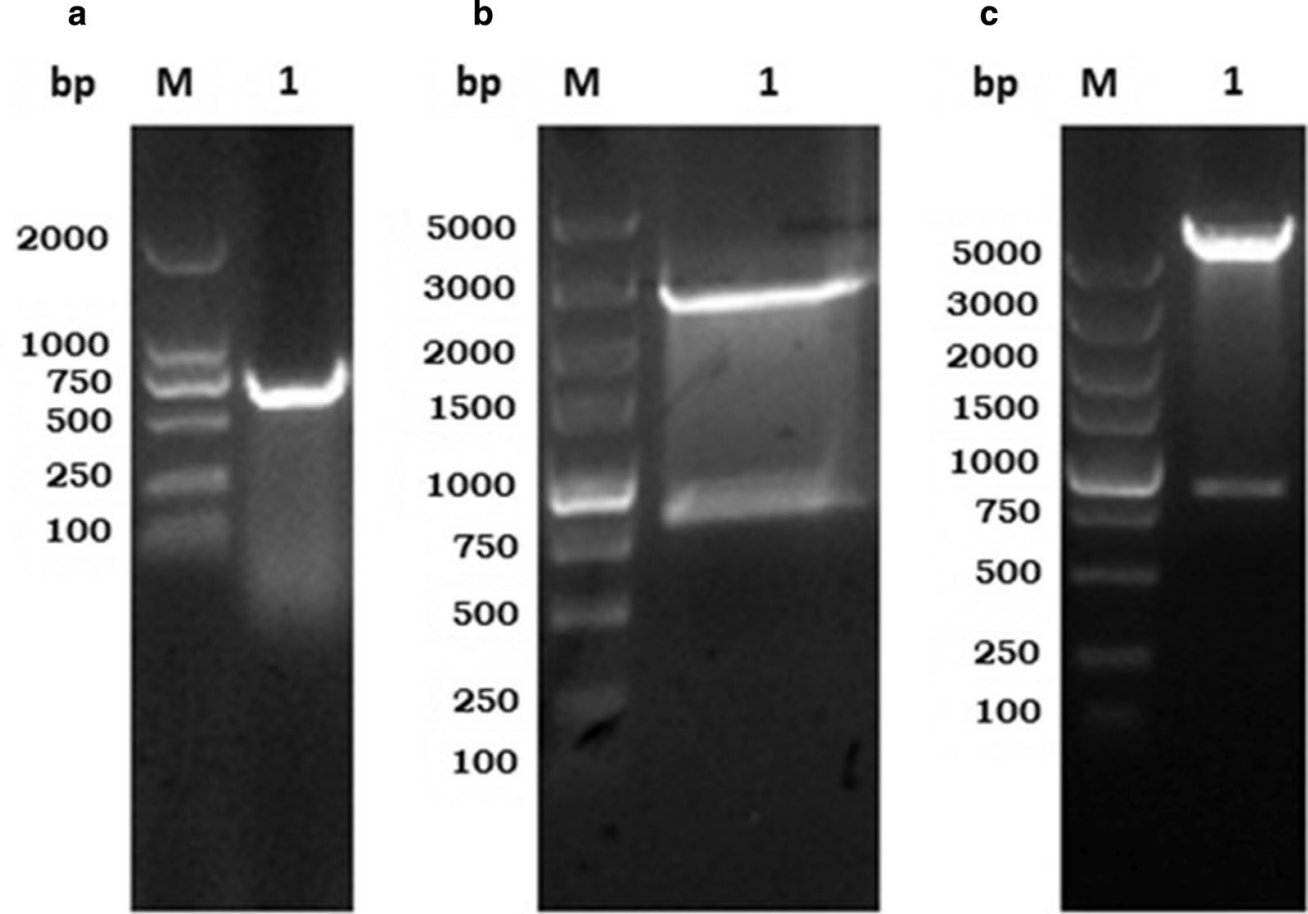
Cloning and expression of HcMTF-12 gene. **a** Gel electrophoresis of HcMTF-12 indicating the 764 bp amplified PCR product. **b** HcMTF-12 cloned into pMD-19T vector and confirmed by restriction enzyme digestion. **c** Recombinant expression plasmid pET-32a (+)-HcMTF-12 verified by restriction digestion with *EcoR*I and *Sac*I

Amplification of the HcMTF-12 gene was successfully performed by PCR from *H. contortus* cDNA with specific primer pairs with the fragment detected at the desired size of 764 bp (Fig. 1). The product was subsequently cloned and confirmed as recombinant plasmid pMD-19T-HcMTF-12 by endonuclease enzymatic digestion with *EcoR*I and *Sac*I (Fig. 1). Further validation was carried out by successful generation of recombinant expression plasmid pET-32a (+)-HcMTF-12 on 1% agarose gel electrophoresis, and sequencing confirmed its correct insertion into accurate reading frame (Fig. 1).

### Sequence analysis using bioinformatics tools

**Fig. 2:**
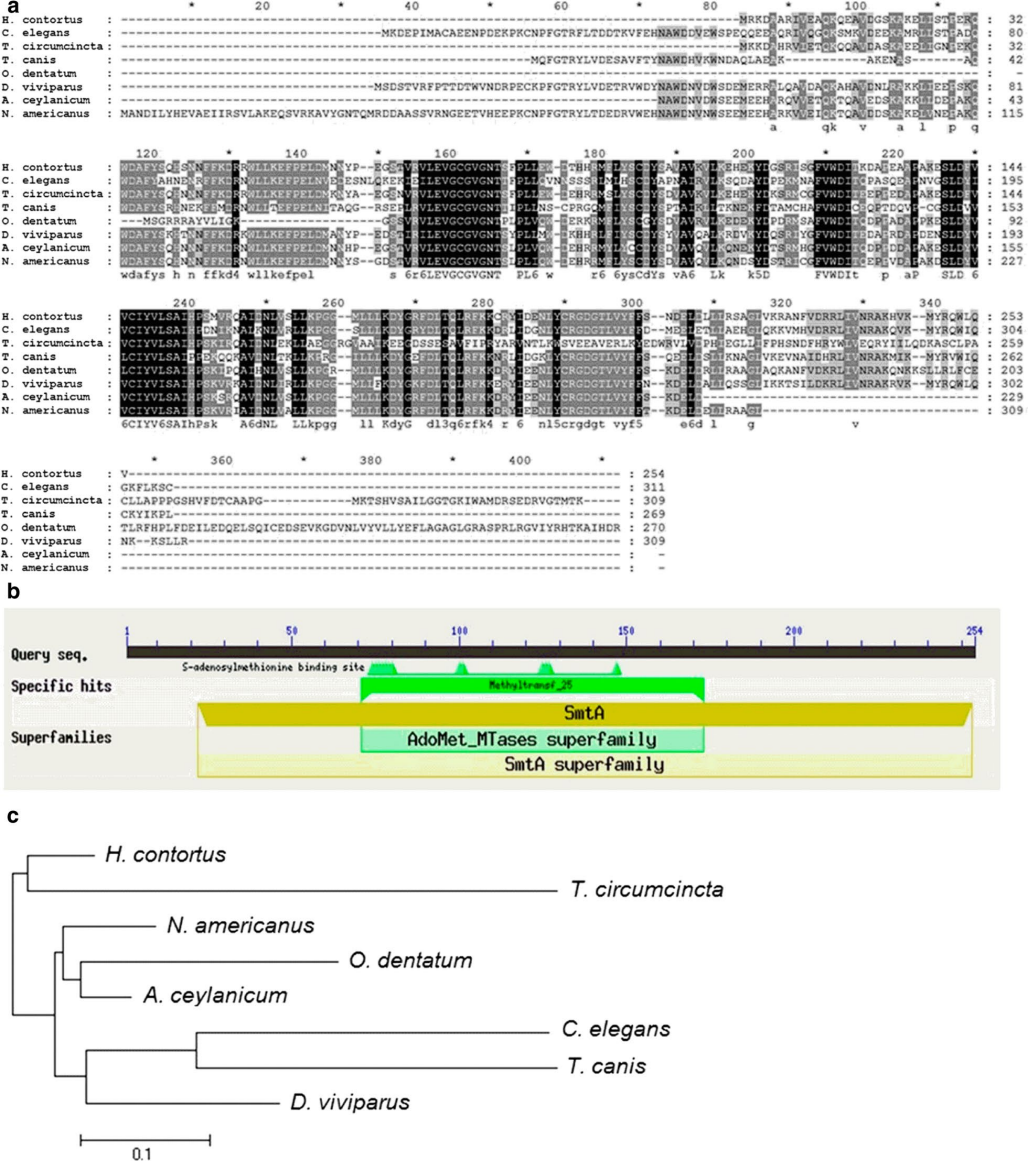
Multiple alignment of amino acid sequence of HcMTF-12. **a** The amino acid sequence of HcMTF-12 aligned with other known methyltransferase family of proteins from the NCBI database, depicted identity with *T. circumcincta* (82%), *A. ceylanicum* (79%), *N. americanus* (78%), *O. dentatum* (77%), *D. viviparus* (74%), *C. elegans* (60%) and *T. canis* (59%). **b** Putative conserved domain. **c** Phylogenetic analysis of the relationships among the amino acid sequences of HcMTF-12 and known similar sequences by minimum evolution

The nucleotide and translated amino acid sequences of HcMTF-12 were analyzed using BLASTx and BLASTp, and compared with known protein sequences from other parasite species available on GenBank. Analysis of the amino acids sequences showed considerable identity (82%) with *Teladorsagia circumcincta* methyltransferase domain protein (GenBank: PIO70527.1), 79% with *Ancylostoma ceylanicum* SCP-like protein (GenBank: EPB79002.1), 78% with *Necator americanus* methyltransferase domain protein (GenBank: XP_013304755.1), 77% with *Oesophagostomum dentatum* methyltransferase domain protein (GenBank: KHJ75858.1), 74% with *Dictyocaulus viviparus* methyltransferase domain protein (GenBank: KJH40840.1), 60% with *Caenorhabditis elegans* methyltransferase-like protein (GenBank: NP_001040827.1) and 59% with *Toxocara canis* methyltransferase-like protein 2-A (GenBank: KHN87041.1) (Fig. 2). The distinctive characteristics of HcMTF-12 were verified as S-adenosyl-l-methionine binding sites in its structure (Fig. 2). The target sequence was further analyzed by multiple alignment with a number of published sequences and determined that the cloned ORF of HcMTF-12 belongs to the *H. contortus* Adenosyl-methionine superfamily. The phylogenic tree analysis using MEGA 5.10 software indicated that HcMTF-12 was accurately allied with MTF of homologous protein sequences obtained from NCBI and represented as bootstrap values for 1000 replicates (Fig. 2). The bootstrap consensus calculation showed high amino acid sequence similarity between HcMTF-12 and the available data on GenBank for *T. circumcincta*, *A. ceylanicum*, *N. americanus*, *O. dentatum*, *D. viviparus*, *C. elegans* and *T. canis*.

### rHcMTF-12 protein expression and purification

**Fig. 3:**
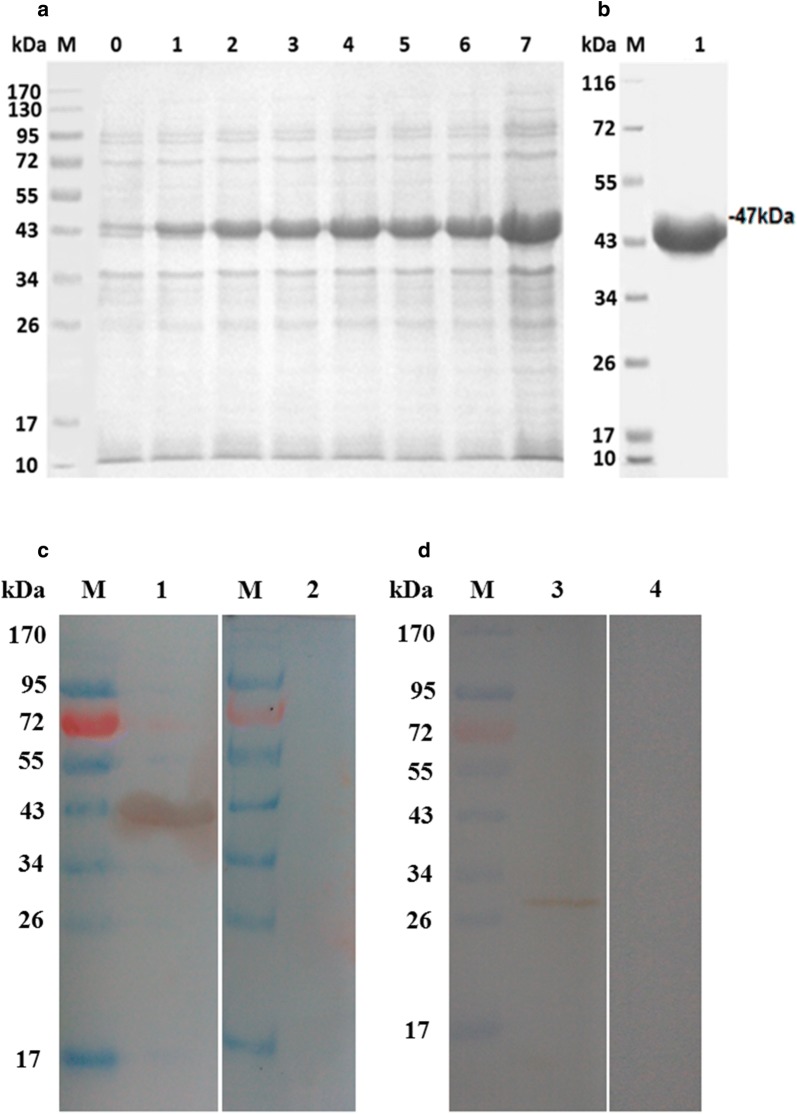
Expression, purification and immunoreactivity of rHcMTF-12. **a** Lane M: standard protein molecular weight marker; Lane 0: recombinant expression vector before induction; Lane 1–7: protein expression induced with 1 mM IPTG. **b** Lane 1: purified rHcMTF-12 protein resolved on SDS-PAGE. **c** Lane M: molecular weight protein ladder; Lane 1: purified rHcMTF-12 (transferred to a membrane probed with serum from goat infected with *H. contortus*); Lane 2: membrane probed with normal goat serum as control. **d** Lane 3: total ES proteins of *H. contortus* probed with antibodies from SD rats immunized with rHcMTF-12; Lane 4: membrane probed with normal rat sera as control

The recombinant plasmid rHcMTF-12 inserted into *E. coli* strain BL21was expressed as a fusion protein after 0.8 mM IPTG induction and purified by chromatography on a Ni-NTA super column. The purified product was electrophoresed on a 12% SDS-PAGE and stained with Coomassie brilliant blue (Fig. 3). The rHcMTF-12 protein was expressed at a molecular weight of 47 kDa rather than calculated mass of 29 kDa due to the extra-size of the vector protein (pET32a), which was approximately 18 kDa fused with the target protein (Fig. 3).

### Antibodies detected by western blot analysis

Immunoblotting was utilized to detect polyclonal antibodies against rHcMTF-12, which showed that rHcMTF-12 could be detected with antibodies in sera of goats infected with *H. contortus* (Fig. 3). The native MTF-12 antigen in whole soluble extracts of *H. contortus* was distinguished with antibodies raised in SD rats immunized with rHcMTF-12 and the presence of a single band indicated that these antibodies had only specificity against the MTF-12 protein of *H. contortus* (Fig. 3). However, sera taken from un-immunized goats/rats did not show any antibodies against the protein.

### Detection of rHcMTF-12 on the surface of goat PBMCs

**Fig. 4:**
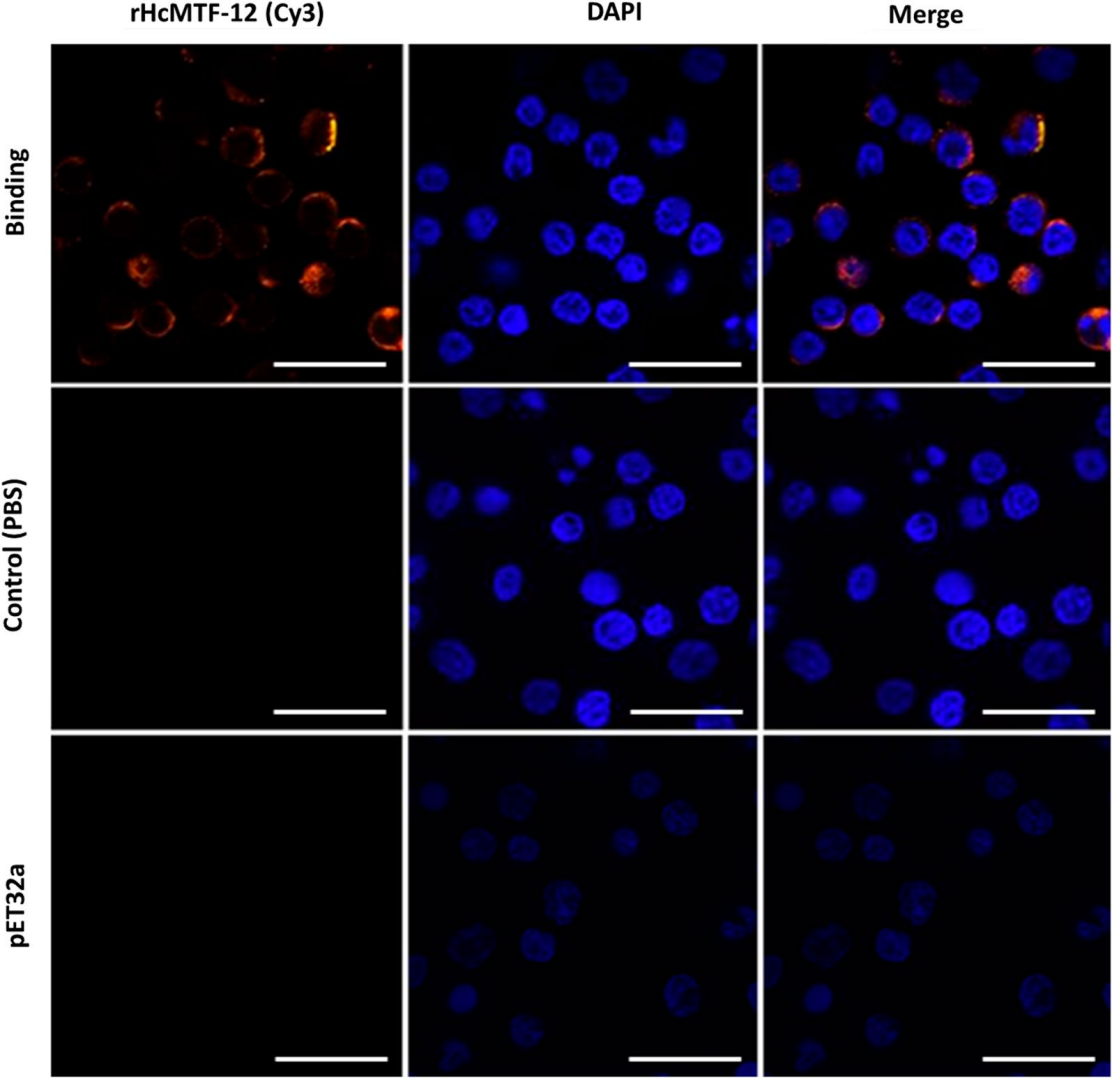
Binding of rHcMTF-12 protein with goat PBMCs. The cells were incubated with rat sera anti-rHcMTF-12 IgG, negative rat IgG or anti-pET32a protein IgG as the primary antibody followed by Cy3-labeled goat anti-rat IgG as the secondary antibody. Red fluorescence on surface of cells showed target protein staining (Cy3) and cell nuclei were visualized by DAPI (blue). No fluorescence was observed in the PBS control or pET32 control groups. *Scale-bars*: 10 μm

The interactions of rHcMTF-12 with host immune cells were detected by immunofluorescence assay (IFA) according to previously described procedures [17]. Results showed red fluorescence at the periphery of incubated PBMCs thus confirming Cy3-labeled rHcMTF-12 binding in the treatment group along with the DAPI labelled cell nuclei displaying in blue color. However, in the control groups, no red visualization was detected either in cells incubated with pET32a control protein or the PBS control. These results indicate that rHcMTF-12 could attach on PBMCs surface in shape of surface ligand complex and suppress or modulate the immune functions of host PBMCs (Fig. 4).

### Distribution of MTF-12 protein within the worm

**Fig. 5:**
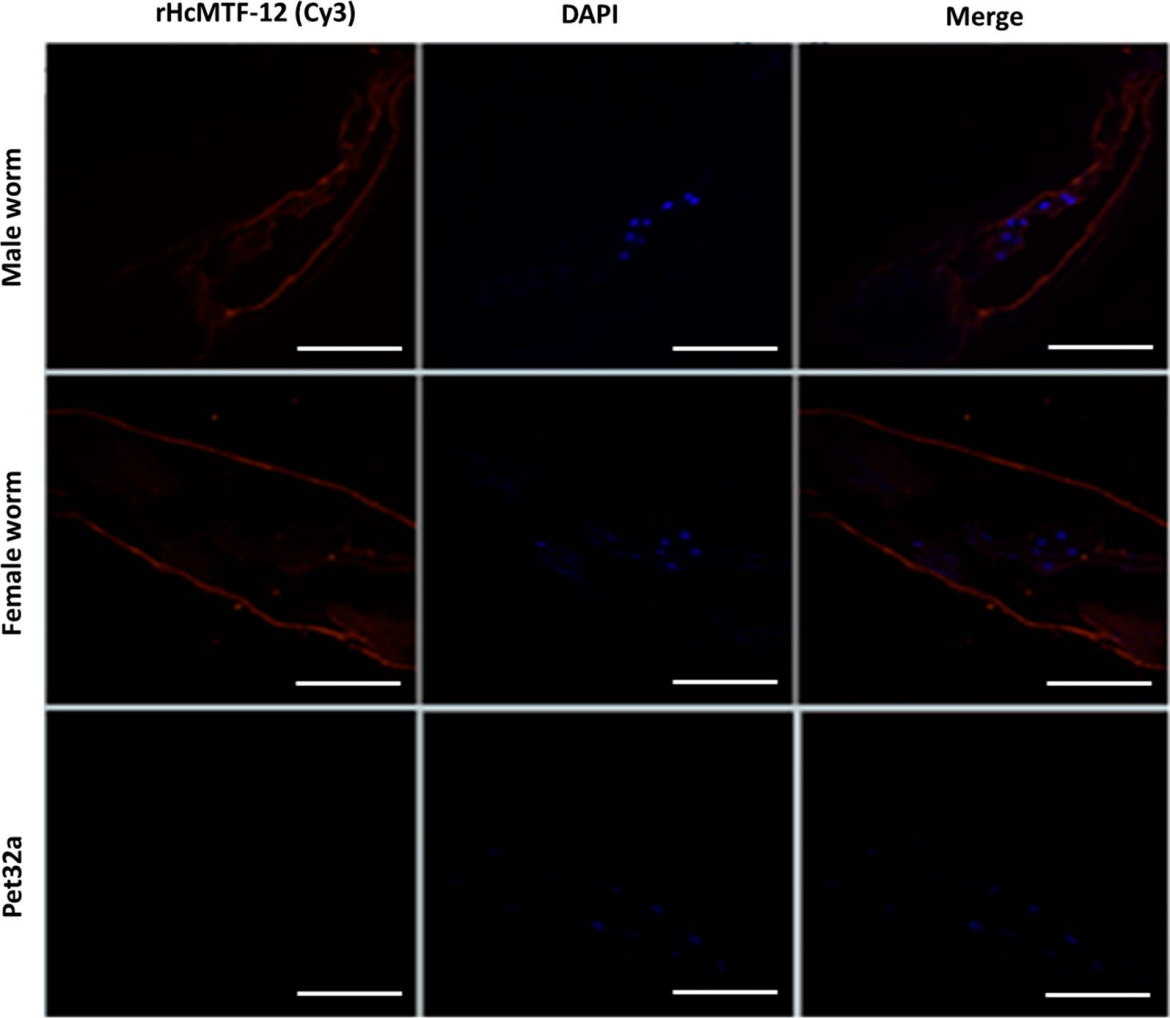
Localization of MTF-12 protein within worm sections. Immunohistochemically, the MTF-12 protein localizes in the cuticle as well as within the pharynx and intestinal region of *H. contortus* male and female adult worms. The red color indicates the target protein stained with Cy3 and blue dots indicate the localization of nuclei stained with DAPI. No red fluorescence was detected in the control panel. *Scale-bars*: 100 μm

As shown in Fig. 5, adult male and female worms were used to detect the target protein within partial body sections (not whole body length) by immunohistochemical assay. The distribution of the native MTF-12 protein within the worm was indicated as red fluorescence in the cuticle as well as within the pharynx and intestinal region in treatment groups. However, in control sections, no protein localization was detected (Fig. 5).

### rHcMTF-12 mediated cytokine expression of goat PBMCs

**Fig. 6:**
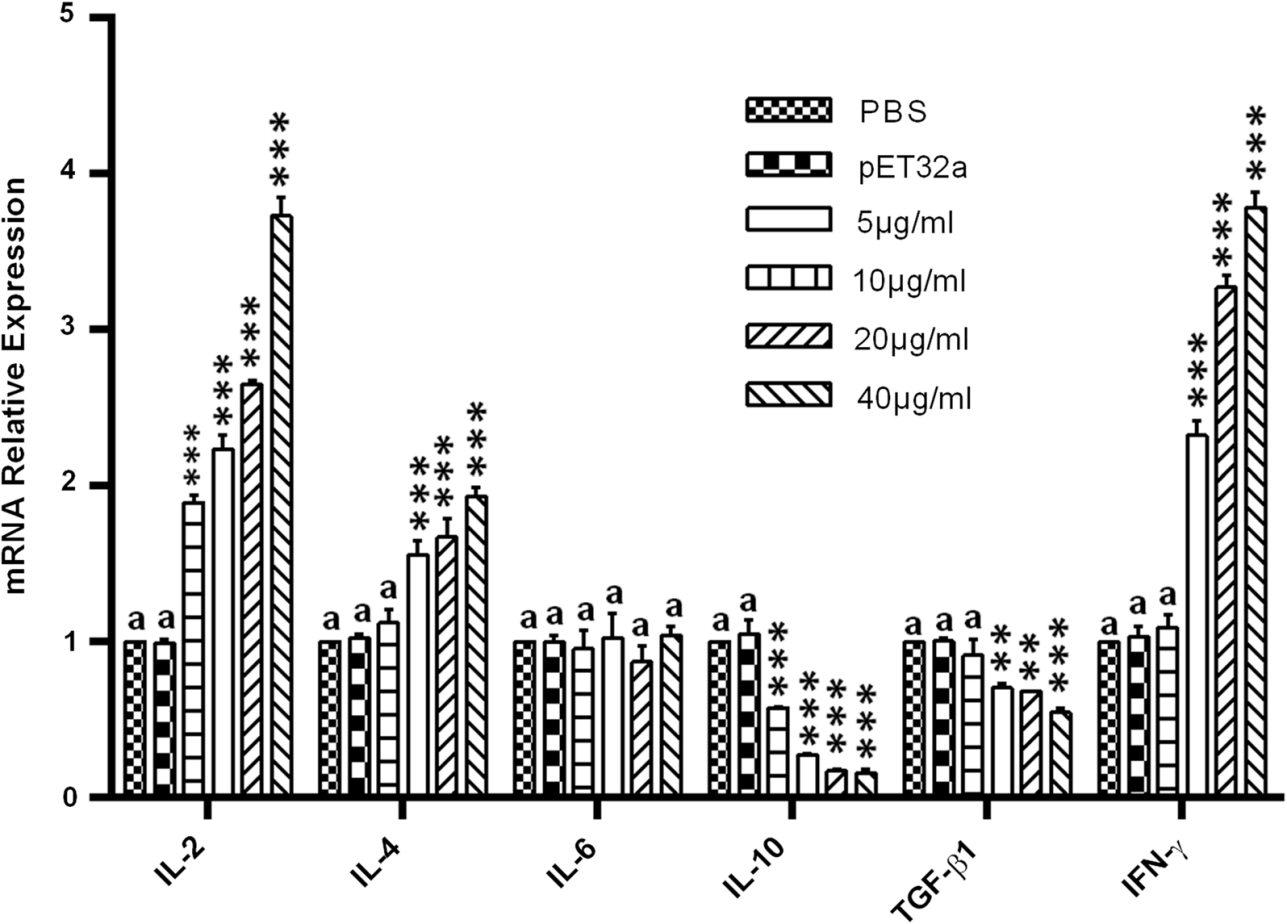
Relative expression of multiple cytokines in goat PBMCs stimulated by rHcMTF-12. Cells were incubated with rHcMTF-12 for 48 h, and the mRNAs encoding IL-2, IL-4, IL-6, IL-10, TGF-β1 and IFN-γ were quantified by real-time PCR. The significant level was set at *P < 0.05, **P < 0.01 and ***P < 0.001 compared to the untreated group (control). PBMC used for all replicates of distinct treatments in each experimental repetition were derived from the same goat. Data are representative of three independent experiments

The mRNA levels of cytokines in PBMCs cultured with rHcMTF-12 protein were evaluated, and the results showed that transcriptions of interleukin-2 (IL-2) (ANOVA: *F*_(5, 12)_ = 243.1, *P* < 0.0001), IL-4 (ANOVA: *F*_(5, 12)_ = 28.31, *P* < 0.0001) and IFN-γ (ANOVA: *F*_(5, 12)_ = 266.5, *P* < 0.0001) were augmented at a considerable level in a dose-dependent manner. The secretions of TGF-β1 (ANOVA: *F*_(5, 12)_ = 18.98, *P* < 0.0001) and IL-10 (ANOVA: *F*_(5, 12)_ = 117.2, *P* < 0.0001) were significantly decreased in PBMCs incubated with rHcMTF-12 in comparison with the pET32a control group and PBS control group. However, IL-6 was not significantly different (ANOVA: *F*_(5, 12)_ = 0.410, *P* < 0.833) when compared to the control group (Fig. 6).

### rHcMTF-12 downregulated multiplication of goat PBMCs

**Fig. 7:**
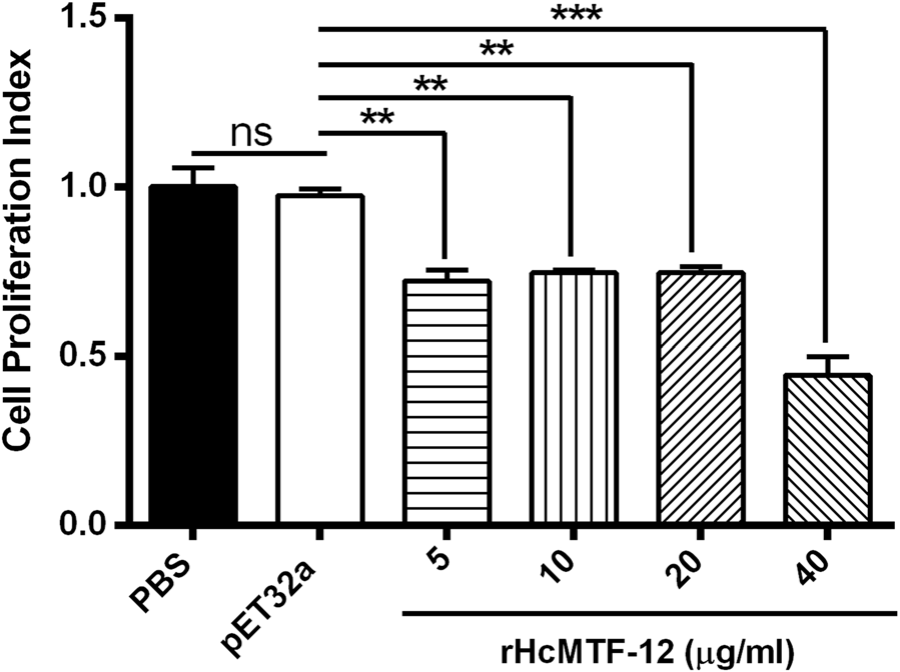
Decreased level of cell proliferation by rHcMTF-12 *in vitro*. Cells were simulated with ConA and treated with control buffer (PBS), pET32a protein and various concentrations of rHcMTF-12 protein for 72 h. A proliferation test was conducted by CCK-8 and values were measured at 450 nm wavelength (OD_450_) using microplate spectrophotometer. Cell proliferation index was calculated by considering the OD_450_ values in the controls as 100%. PBMC used for all replicates of distinct treatments in each experimental repetition were derived from the same goat. The data are expressed as the mean ± standard error (SE) of three independent experiments. **P < 0.01, ***P < 0.001. *Abbreviation*: ns, non-significant

The anti-proliferative effect of rHcMTF-12, in comparison with the PBS control and pET32a protein groups, was estimated using a cell counting kit (CCK8). Our results highlighted that PBMCs activated with ConA and treated with rHcMTF-12 protein downregulated PBMCs multiplication efficiency at 5 µg/ml (ANOVA: *F*_(5, 12)_ = 30.44, *P* = 0.0014), 10 µg/ml (ANOVA: *F*_(5, 12)_ = 30.44, *P* = 0.0012), 20 µg/ml (ANOVA: *F*_(5, 12)_ = 30.44, *P* = 0.0013) and 40 µg/ml (ANOVA: *F*_(5, 12)_ = 30.44, *P* = 0.0002) multiple protein concentrations dose-dependently (Fig. 7). However, cell proliferation was significantly unchanged between the pET32a group and the control buffer group (ANOVA: *F*_(5, 12)_ = 30.44, *P* = 0.692).

### rHcMTF-12 promoted migration efficiency of PBMCs

**Fig. 8:**
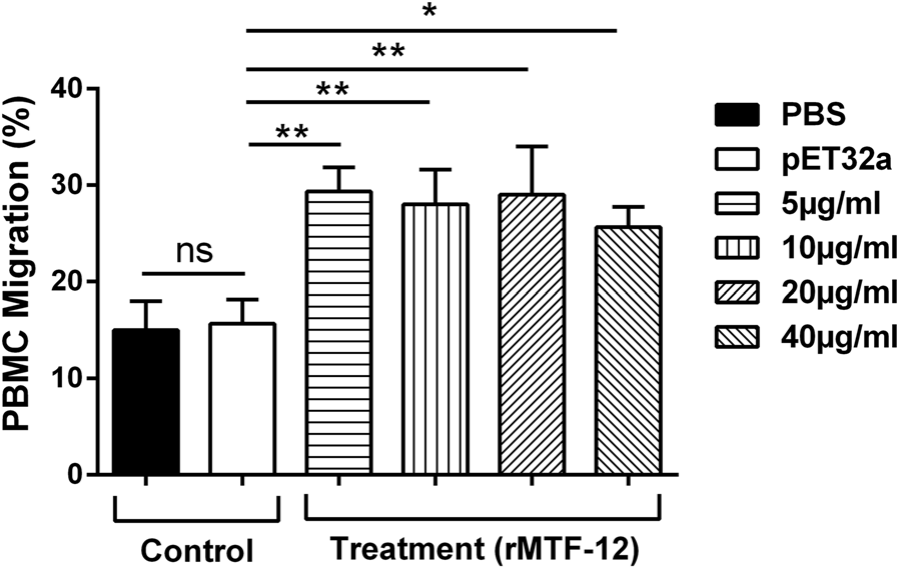
Effects of the various concentrations of rHcMTF-12 on PBMC migration *in vitro*. Cells were treated with control buffer, pET32a protein and multiple concentrations of rHcMTF-12. The migration percentage was determined randomly. The difference between the mean values was calculated using ANOVA. PBMC used for all replicates of distinct treatments in each experimental repetition were derived from the same goat. Data are representative of three independent experiments. *P < 0.05, **P < 0.01. *Abbreviation*: ns, non-significant

The change in migratory efficiency of host PBMCs in response to rHcMTF-12 was assessed by the percentile of shifted PBMCs through the Millipore polycarbonate membrane into the lower chamber. Our results revealed that no significant cell movement was achieved in the control buffer and protein (pET32a) group (ANOVA: *F*_(5, 12)_ = 12.51, *P* = 0.783). However, in treatment groups of 5 µg/ml (29.33 ± 1.453%) (*P* = 0.003), 10 µg/ml (28.00 ± 2.082%) (*P* = 0.008), 20 µg/ml (29.00 ± 2.887%) (*P* = 0.0014) and 40 µg/ml (25.67 ± 1.202%) (*P* = 0.014) of rHcMTF-12, a significant percentage of cells migrated through the membrane (Fig. 8).

### rHcMTF-12 mediated NO production in goat PBMCs

**Fig. 9:**
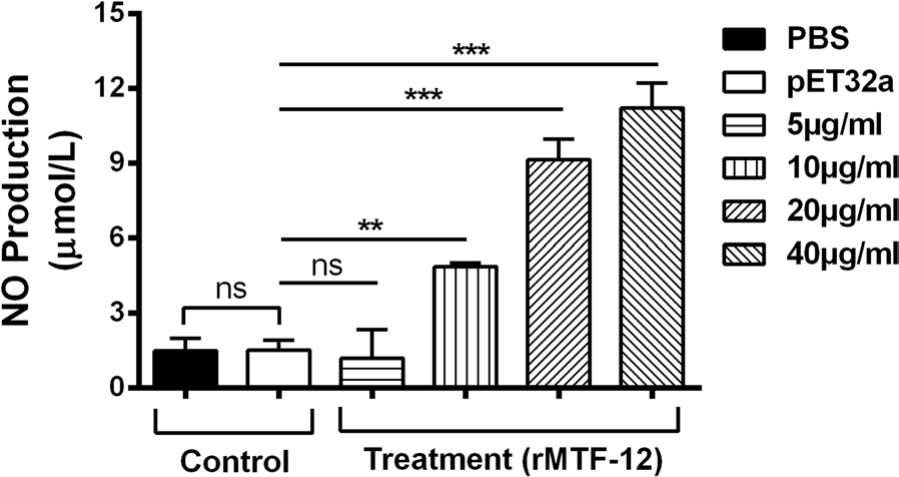
Measurement of the intracellular nitric oxide production by goat PBMCs *in vitro*. Nitric oxide concentration in the supernatant of cell cultures was performed according to the instructions of Griess assay Total Nitric Oxide Assay Kit. PBMC used for all replicates of distinct treatments in each experimental repetition were derived from the same goat. The data are presented as the mean ± standard error (SE) of three independent experiments. **P < 0.01, ***P < 0.001. *Abbreviation*: ns, non-significant

Using the Griess assay, no significant NO production occurred between control group and 5 µg/ml protein concentration (ANOVA: *F*_(5, 12)_ = 98.41, *P* = 0.716). The rHcMTF-12 treatment groups showed a significant difference in NO production by PBMCs at 10 µg/ml (ANOVA: *F*_(5, 12)_ = 98.41, *P* < 0.001), 20 µg/ml (ANOVA: *F*_(5, 12)_ = 98.41 *P* < 0.001) and 40 µg/ml (ANOVA: *F*_(5, 12)_ = 98.41, *P* < 0.001) when compared to the control group. Meanwhile, there was no significant difference between the PBS and pET32a groups (ANOVA: *F*_(5, 12)_ = 98.41, *P* = 0.682) (Fig. 9).

## Discussion

S-Adenosyl methionine (SAM)-dependent methyltransferases genes have been implicated in numerous biological processes through various cellular pathways, which regulate gene expression and/or functions [28]. However, nothing is known about methyltransferases regulation of immunity during host–parasite interactions at the cellular level. Here, we report the host cell binding protein of *H. contortus* parasite (HcMTF-12) and its immuno-regulatory characteristics with host PBMCs *in vitro* for the first time.

Previously, it was reported that a large class of SAM-dependent methyltransferases shared a conserved catalytic domain structurally in interaction with S-adenosylmethionine as a common cofactor [29]. In addition, nine AdoMet-dependent methyltransferases had been found to have same folding arrangement in a central parallel β-sheet encircled by α-helicals [30–32], and a large number of AdoMet-dependent methyltransferases shared conserved sequence motifs in their three-dimensional structure [33–35]. In this study, we cloned a SAM-dependent methyltransferase gene from *H. contortus* (HcMTF-12), which showed highly similar sequence identity and structural characteristics among various sequences of methyltransferases enzymes from other parasitic species. The size of the native HcMTF-12 was *c.*29 kDa in somatic extracts of *H. contortus* detected by the antibodies of rat against rHcMTF-12 and the molecular weight of rHcMTF-12 was *c.*47 kDa. Apart from the Tag peptides of 18 kDa of the vector, the size of the rHcMTF-12 was identical to that of the native one. Immunoblotting showed that rHcMTF-12 was identified with sera from experimentally infected with *H. contortus* goats. The anti-rHcMTF-12 antibodies used to probe the whole soluble proteins of *H. contortus* produced only a single band, which indicated that these antibodies did not show cross-reactivity with other proteins in this parasite. Our results suggest that these antibodies had specificity against the MTF-12 protein of *H. contortus*. These results indicate that the native MTF-12 could enter into the circulatory system of the goat and be recognized by the host immune system.

The ES proteins are released at the host–parasite interface and hence the protein-cell interaction is the primary requirement for immune functions [25]. A previous study demonstrated that methyltransferase from *Plasmodium falciparum* localized in membrane structures where it proposed to play an important role in parasite membrane biogenesis [36]. In the present study, we detected HcMTF-12 protein interaction on the surface of host PBMCs using IFA. Its localization was detected within the parasite structure by immunohistochemical analysis, which confirmed that HcMTF-12 protein was mainly located in the cuticle as well as within the pharynx and intestinal region.

T helper cells 1 (Th1) and Th2 along with inflammatory responses are linked with production of numerous cytokines that play vital anti-parasitic roles against helminths particularly *H. contortus* infection [37]. Previously, it was noted that a type-2 immune response was responsible to helminth infections, which were usually associated with IL-10, IL-5 and IL-4 secretions [38]. In addition, interleukin 10 (IL-10), a key element generated through inducible T regulatory cells (Treg), was responsible to prevent production of allergic Th2 responses [39]. In our study, IL-4 secretion by PBMCs in response to rHcMTF-12 protein was increased, while the secretion of the immunosuppressive IL-10 was decreased. This indicates that rHcMTF-12 might be a protective antigen which diminished the effects of Treg cells and induced Th2 humoral responses against *H. contortus* infection.

Apart from humoral responses, cellular immunity is another important mechanism against parasitic infection regulated by Th1 cells which produce IFN-γ [40, 41]. Moreover, progression of Th2 cells in immune responses was found to be negatively regulated by IFN-γ [42]. According to Schallig et al. [43], sheep experimentally infected with *H. contortus* retorted to a cell mediated immune response, which was characterized with high levels of IL-2 and IFN-γ. In the present study, higher expressions of IFN-γ and IL-2 cytokines were detected in goat PBMCs after incubation with rHcMTF-12 protein. In this regard, a higher level of IL-4 and IFN-γ suggested a balanced Th1 and Th2 environment which might either be a collective effect of immune cells (PBMCs) or an individual cell playing a distinctive role; this needs to be investigated further.

Transforming growth factor beta 1 (TGF-β1) is a multifunctional interleukin which differentially operates several immune system events by various cell types and potentially regulates many biological functions [44]. Moreover, it was suggested that TGF-β1 also played a dynamic role in inhibition of cell multiplication and stimulation of programmed cell death of numerous immune cell subsets [45]. The production of TGF-β1 after incubation of goat PBMCs with rHcMTF-12 increased at a significant level, which might be another mechanism of rHcMTF-12 antigen to promote immune evasion at the parasite-host interface.

Interleukin 6 (IL-6), a pro-inflammatory cytokine, was released in reaction to infection and contributed in the host defense mechanism by generation of an acute stage immune response. In this study, the IL-6 did not show any changes indicating that rHcMTF-12 protein could not stimulate IL-6 production.

Modulation of immune responses during the infection process is also linked with active stimulation and recruitment of eosinophils and other lymphocytes by helminths, resulting in stability of worms within the host [46, 47]. Previously, we determined stimulatory roles of HcESPs collectively and individually in the case of rHcftt-2 and rHcES-24 to induce the migration of goat PBMCs [20, 48, 49]. Here, we also found that rHcMTF-12, as an interacting protein of HcESPs, showed significant enhancement ability to the migration of host PBMCs. These results indicate that rHcMTF-12 protein might be an active contributor of HcESPs in the modulation of PBMCs migration. However, the underlying mechanism involved behind this strategy needs to be further investigated.

Proliferation of immune cells plays an important role in regulation of immune responses during helminth infection [50, 51]. Previously, we found a suppressive effect of various *H. contortus* ES proteins on PBMC multiplication [20, 48, 49, 52]. In accordance with earlier studies, the present study revealed that rHcMTF-12 could also significantly inhibit the proliferation of goat PBMCs.

Previous molecular approaches suggested that protein methyltransferase is actively involved in regulation of NO release by endothelial cells [53]. NO was involved in non-specific host resistance and cytotoxic activities against a variety of parasitic infections, and its release could be enhanced by IFN-γ [54, 55]. The results from the present study implicate that rHcMTF-12 could stimulate the NO production. This result together with the increased level of IFN-γ, suggest that rHcMTF-12 might be an important contributor in the host defense mechanism against *H. contortus* infection.

## Conclusions

We first cloned and characterized HcMTF-12, an important ES antigen of *H. contortus*. Our results show that rHcMTF-12 protein could bind to the host immune cells and modulate a variety of immune functions, including upregulation of IFN-γ, IL-4 and IL-2, and enhancing PBMCs migratory activity and NO production. However, IL-10 and TGF-β1 production and cell multiplication were suppressed at different rHcMTF-12 protein doses. These results indicate that the HcMTF-12 protein plays a modulatory role on multiple functions of host PBMCs. However, the underlying regulatory mechanisms of HcMTF-12 *in vivo* during host–parasite interactions need to be explored in future studies.

## Supplementary information

**Additional file 1: Table S1.** Primers sequences for real-time PCR. **Table S2.** Reagents used for real-time PCR. **Table S3.** Thermal cycling conditions for real-time PCR.

## Data Availability

The datasets supporting the conclusions of this article are included within the article and in Additional file 1: Tables S1–S3.

## Abbreviations

MTF-12: methyltransferase-type 12
LB: Luria Bertani medium
IPTG: isopropyl-Bd-thiogalactopyranoside
Ni-NTA: Ni^2+^-nitrilotriacetic acid
SDS-PAGE: sodium dodecyl sulfate polyacrylamide gel electrophoresis
PVDF: polyvinylidene difluoride
IgG-HRP: horseradish peroxidase labeled immunoglobulin G
DAB: 3, 3-diaminobenzidine tetrahydrochloride
DEPC: diethyl pyrocarbonate
OCT: optimal cutting temperature
BSA: bovine serum albumin
PBMCs: peripheral blood mononuclear cell
PBS: phosphate buffered saline
RPMI 1640: Roswell park memorial institute 1640 culture media
FBS: fetal bovine serum
con A: concanavalin A
Cy3: cyanine dyes 3
DAPI: 2-(4-amidinophenyl)-6-indolecarbamidine dihydrochloride
Th1: helper T cell 1
Th2: helper T cell 2
IL-10: interleukin-10
IL-6: interleukin-6
IL-4: interleukin-4
IL-2: interleukin-2
IFN-γ: interferon-γ
TGF-β1: transforming growth factor-β1
IL-17: interleukin-17
