# Supplementary material for: Identification of a novel methyltransferase-type 12 protein from Haemonchus contortus and its effects on functions of goat PBMCs
Source: Parasit Vectors. 2020 Mar 30;13:154. doi: 10.1186/s13071-020-04028-y (PMC7106832; doi:10.1186/s13071-020-04028-y)
Supplement: Supplementary file 1 — Additional file 1: Table S1. Primers sequences for real-time PCR. Table S2. Reagents used for real-time PCR. Table S3. Thermal cycling conditions for real-time PCR. [file 13071_2020_4028_MOESM1_ESM.docx]

**Additional file 1: Table S1. Primers sequences for real-time PCR.**

| Gene Name | Primer Sequence (5’-3’) | gene length (bp) | Amplification efficiency  (%)* | Correlation  coefficients (r^2^) |
| --- | --- | --- | --- | --- |
| β-actin | CACCACACCTTCTACAAC | 106 | 95.41 | 0.9991 |
|  | TCTGGGTCATCTTCTCAC |  |  |  |
| IL-2 | CAAACGGTGCACCTACTTCA | 115 | 96.75 | 0.9985 |
|  | AGCTTGAGGTTCTCGGGATT |  |  |  |
| IL-4 | GTACCAGCCACTTCGTCCAT | 148 | 98.73 | 0.9994 |
|  | GCTGCTGAGATTCCTGTCAA |  |  |  |
| IL-6 | CGTCGACAAAATCTCTGCAA | 149 | 98.45 | 0.9997 |
|  | TTCCCTCAAACTCGTTCTGG |  |  |  |
| IL-10 | CCTTGTCGGAAATGATCCAG | 150 | 98.68 | 0.9993 |
|  | AGGGCAGAAAACGATGACAG |  |  |  |
| TGF-β1 | GAACTGCTGTGTTCGTCAGC | 126 | 98.98 | 0.9996 |
|  | TCCAGGCTCCAGATGTAAGG |  |  |  |
| IFN-γ | GAACGGCAGCTCTGAGAAAC | 131 | 98.02 | 0.9982 |
|  | GGTTAGATTTTGGCGACAGG |  |  |  |

* Amplification efficiency (%) = (10^-1/slope^ -1) ×100

**Additional file 1: Table S2. Reagents for real-time PCR.**

| **Reagent** | **Volume** |
| --- | --- |
| 2×ChamQSYBR qPCR Master Mix | 10.0 µl |
| Primer 1（10µM） | 0.4 µL |
| Primer 2（10µM） | 0.4 µL |
| 50×ROX Reference Dye 2（50×） | 0.4 µL |
| cDNA | X µL |
| ddH_2_O | To 20.0 µL |

**Additional file 1: Table S3. Cyclic conditions for real-time PCR.**

| Stage 1 | Pre-degeneration | Reps: 1 | 95 °C | 30 sec |
| --- | --- | --- | --- | --- |
| Stage 2 | Circular reaction | Reps: 40 | 95 °C | 10 sec |
|  |  |  | 60 °C | 30 sec |
| Stage 3 | Melt curve | Reps: 1 | 95 °C | 15 sec |
|  |  |  | 60 °C | 60 sec |
|  |  |  | 95 °C | 15 sec |
